# A Pilot Group Visit Series to Empower Opportunity Youth: Building Skills for Managing Pain, Anxiety, and Distress

**DOI:** 10.3390/healthcare14213567

**Published:** 2026-10-22

**Authors:** Katherine Shafto, Catherine Justice, Jadyn Knox, Peter Scal, Tegan J. Reeves, Kara Lawrence, Susan Haddow, Jacinta Rebang, Arti Prasad

**Affiliations:** 1 Hennepin Healthcare Systems, Minneapolis, MN 55415, USA; justicecat@gmail.com (C.J.); reevest@rhodes.edu (T.J.R.); susan.haddow@hcmed.org (S.H.); rebang.achu@yahoo.com (J.R.); aprasad@salud.unm.edu (A.P.); 2 Bakken Center for Spirituality and Healing, University of Minnesota, Minneapolis, MN 55455, USA; 3 Hennepin Healthcare Research Institute, Minneapolis, MN 55415, USA; jadynknox2626@gmail.com; 4 Department of Pediatrics, University of Minnesota, Minneapolis, MN 55455, USA; scal0005@umn.edu; 5 Department of Counseling Psychology, Education and Research, The University of Memphis, Memphis, TN 38152, USA; klwrnce4@memphis.edu

**Keywords:** chronic pain, group medical visits, opportunity youth, shared medical appointments, mental health, peer connection

## Abstract

**Highlights:**

**What are the main findings?**

- A pilot 8-week Group Visit Series (GVS) on whole-person approaches to managing pain, anxiety, and distress with 18- to 24-year-old opportunity youth showed high engagement, feasibility, and acceptability.
- In exploratory signals from participant-reported pre- to post-series PROMIS-29 (*n* = 6), 6/6 (100%) participants met a minimal clinical important difference (MCID) threshold for depression and 5/6 (83%) in anxiety.

**What are the implications of the main findings?**

- Opportunity youth at an employment-readiness center responded positively to this GVS, leading to continuation of the series beyond the pilot.
- This pilot GVS offers an example of a group-based program to build skills that support adolescents at high risk of chronic pain and mental health challenges.

**Abstract:**

**Background/Objective**: Adolescents experience stress, anxiety, depression, and chronic pain at rates comparable to adults. Opportunity youth are a unique subset of adolescents/young adults with a higher risk of chronic pain and mental health comorbidities. Group Medical Visits (GMVs) for adults are a well-established model supporting whole-person care for chronic pain and co-incident mental health challenges. Research on GMVs among adolescents is limited. This program evaluation explores the feasibility, acceptability, and sustainability of a chronic pain GMV curriculum adapted for opportunity youth at a residential employment-readiness center. Preliminary signals of program impact were explored as a secondary objective of the study. **Methods**: After completing a chronic pain GMV training program, two pediatricians adapted and piloted the curriculum for opportunity youth through an 8-week Group Visit Series (GVS) offering integrative and lifestyle-based approaches to mental health, stress, and chronic pain. This was a single-site, uncontrolled, mixed-methods pilot program evaluation. Attendance was recorded as a measure of feasibility. A weekly progress sheet captured pre-/post-visit participant feedback, and semi-structured interviews (*n* = 6), conducted one month post-GVS, were examined using rapid analysis to explore acceptability. Sustainability was observed by program continuation beyond the pilot. Exploratory participant-reported outcomes were collected through PROMIS-29 (*n* = 6), PROMIS Positive Affect (*n* = 5), and Patient Global Impression of Change (*n* = 6). **Results**: Fourteen participants enrolled, with nine completing ≥3 visits. Weekly progress sheets demonstrated intra-visit changes from pain, fatigue, and concern to calm. Interview analysis yielded concepts of appreciation for skills learned and the group experience. Sustainability was observed. Six of seven PROMIS-29 domains showed positive signals of improvement, with 6/6 (100%) of respondents meeting a minimal clinical important difference (MCID) threshold for depression and 5/6 (83%) for anxiety. PROMIS-Positive Affect and Patient Global Impression of Change showed positive trends at visit 8. **Conclusions**: This chronic pain and mental health GVS for opportunity youth was found to be feasible, acceptable, and sustainable. Positive trends in participant-reported depression and anxiety scores were also observed. Larger studies beyond program evaluation are needed to confirm these results.

## 1. Introduction

Adolescence represents a developmental stage characterized by increasing independence, physical/emotional/psychological maturation, and transition into adulthood. The prevalence of pain among adolescents mirrors that of the adult population [1,2], with 11–38% of adolescents experiencing chronic pain. Stress, sleep disturbance, depression, anxiety, and social functioning challenges are all reported as coincident with chronic pain, as well as being common during adolescence in the absence of a pain condition [2,3,4].

Adverse childhood experiences and negative life events in childhood and adolescence are associated with greater mental health challenges, such as anxiety and depression [5]. Opportunity youth is a term that describes young people between the ages of 16 and 24 who are disconnected from school and/or work, often having already experienced adverse experiences, and are at high risk of long-term emotional, behavioral, and chronic health conditions [6,7]. Programs that foster peer connection, life skill-building, and vocational-readiness training have shown success in reconnecting these disconnected youth and reducing long-term negative outcomes [6]. Skill-building and peer connection are often incorporated into such programs and prevention efforts, given the recognition that opportunity youth may not have had supportive family, community, school, sports, or extracurricular environments during adolescence and young adulthood.

In adult populations, Group Medical Visits (GMVs) are well established as a model to address a range of conditions, including chronic pain and co-occurring mental health diagnoses, supporting both prevention and management. GMVs among adults also address loneliness and social isolation by leveraging the support of a group of people who have a similar experience, while also taking place within a healthcare context [8,9]. Current, guideline-aligned whole-person and rehabilitative approaches to chronic pain care emphasize the multidimensional nature of chronic pain, offering multiple doorways through which to improve the chronic pain experience and its impact on quality of life [10]. The biopsychosocial–spiritual model is one such contextualization utilized in chronic pain care and GMV programs [11,12,13,14].

The literature regarding GMVs among adolescents, including opportunity youth, is limited, with the model being used for adolescents with Type 1 Diabetes and ADHD [15,16,17], but less so for chronic pain or mental health conditions [18]. Non-medical or school-based group interventions such as adolescent peer support groups [19,20], college-transition groups for young adults with mental health conditions [21], group therapy, and Dialectical Behavioral Therapy groups [22] have all been studied, with evidence of benefit in their respective settings.

Given the lack of GMVs for adolescents, specifically opportunity youth, and the need for connection and life skills-building in this population, a whole-person chronic pain care GMV curriculum for adults was adapted to a Group Visit Series (GVS) for opportunity youth experiencing chronic pain, anxiety, and/or stress. This was part of a larger Minnesota statewide grant-funded initiative to expand access to whole-person pain care through GMVs and support upstream prevention of opioid-use disorder (OUD) and opioid-related harm. The overarching program, led by The Center for Teaching, Learning, and Coordination–Heals on Wheels (TLC-HoW), included partnerships with three organizations (A, B, and C) across Minnesota. The TLC-HoW program trained clinical teams from each organization in the GMV model, utilizing an established whole-person, non-pharmacologic pain care curriculum that has demonstrated feasibility, acceptability, and positive impact among adult participants in both urban and rural healthcare settings [23,24,25,26]. Organization A, whose work is described in this manuscript, included a clinical team working with adolescents and opportunity youth, who recognized that their population would benefit from skills and education to support mental health, chronic pain prevention, and self-management. The objective of this program evaluation is to assess the feasibility, acceptability, and sustainability of implementing this GVS, as well as the preliminary impact of the pilot program for opportunity youth living at a residential employment-readiness center.

## 2. Methods

### 2.1. Study Design and Setting

This program evaluation was a single-site, uncontrolled, mixed-methods pilot that utilized both quantitative and qualitative data to examine Organization A’s GVS. An urban residential employment-readiness center for young adults considered opportunity youth hosted the program. The GVS was advertised as “a program for young people living with chronic pain, anxiety, and distress.” Visits took place in the summer-fall of 2025, lasted 1.5 h each, and occurred weekly at the host site for a total of eight visits. After the third session, the training center’s funding was disrupted, training was suspended, and students were sent home. Seven weeks later, funding was restored, students returned to the center, and training operations resumed. The GVS resumed with session 4 and experienced no further interruptions. All sessions were held at the training center and led by two pediatricians, one from Organization A and the other affiliated with the employment-readiness center. Students were excused from their scheduled training activities to attend the GVS, but attendance was not mandatory. Students and their trade instructors could prioritize employment training activities, in which case the student did not attend that GVS session. Students were given gift cards and/or self-care tools (~$10 per participant/week) to support participation in the program and skills taught in the GVS.

### 2.2. Curriculum Adaptation

Organization A’s physician and interprofessional team and the host site’s pediatrician participated in the TLC-HoW program’s 10-week, 2 h/week virtual chronic pain GMV training [26]. After the training, the two pediatricians adapted the curriculum to a format suited for opportunity youth and refined it iteratively throughout the GVS. The TLC-HoW curriculum was uniquely tailored to the developmental stage and setting, focusing on skills to prevent and/or manage anxiety, distress, and chronic pain. Table 1 shows the original adult GMV curriculum topics and the adaptation for Organization A’s GVS. Beyond these adjustments in the content and sequencing of topics, other adaptations were made to increase experiential elements, involve local subject-matter experts, promote participant discussion, and expand the focus beyond chronic pain management to include anxiety and stress more generally. Each session included a grounding practice, welcome, introductions, a question of the week, break, teaching/discussion, movement or mind–body skill, and wrap-up with a take-away from the session.

### 2.3. Participant Recruitment

Recruitment began when the center’s census was approximately 200 students. Participants learned about the program through general announcements, a lunchtime promotional event in the cafeteria, and direct outreach by center staff to individual students. The number of eligible students who became aware of the GVS cannot be determined because records were not kept of how many students were reached through these varied recruitment avenues. The program was described as an 8-week group activity where students could learn and practice ways to reduce chronic pain, anxiety, and distress, and improve their health without medication. Participation was open to all students aged 18–24 who expected to remain at the center for the full 8 weeks of the program and had no schedule conflicts with the sessions. Students who expressed interest were enrolled without having to endorse a prior diagnosis of chronic pain, mental health conditions, or any qualifying symptoms. Exclusion criteria were age < 18 years, uncontrolled mental health or addiction diagnosis, or unstable medical/psychiatric condition. Participants signed a confidentiality agreement and consent forms before participating in the GVS, consenting to: (1) voluntary group discussion of personal health information; (2) respecting the confidentiality of group members; and (3) de-identified participant-reported data being shared and analyzed for program evaluation.

### 2.4. Outcomes

Program feasibility was examined through successful implementation of the eight-session GVS with the adapted curriculum for opportunity youth at the employment-readiness center. Additional feasibility criteria included attendance at each session of ≥6 participants for at least 75% of sessions and a weekly progress sheet completion rate of ≥90%. These thresholds were determined based on prior work and program evaluations of GMVs for adults with chronic pain [23]. Participant demographics and attendance were recorded. Enrollment was defined as attending ≥1 visit; minimum participation as attending ≥3 visits. These distinctions were also previously utilized in programs that preceded the TLC-HoW initiative [23,24].

Program sustainability was explored through observation of ongoing host site engagement, student interest, and attendance in subsequent GVS beyond the pilot.

Program acceptability was evaluated through qualitative data from a weekly progress sheet and voluntary post-GVS semi-structured interviews, both described below. The weekly progress sheet specifically asked, “What did you enjoy most about the session?” and “What are your suggestions for improvement?” The post-GVS interviews included similar open-ended questions.

Assessment of the potential preliminary program impact on participants was explored through the aforementioned qualitative data and several validated instruments. The Patient-Reported Outcomes Measurement Information System (PROMIS-29 v.2.1) was administered at visits 1, 4, and 8 of the GVS, measuring 7-day symptom severity for seven discrete domains—Physical Function, Anxiety, Depression, Fatigue, Sleep Disturbance, Ability to Participate in Social Roles and Activities, and Pain Interference—with a single Pain Intensity item [27,28]. The PROMIS-Positive Affect (Short Form v.1.0), administered at visits 1 and 8, is a 15-item survey assessing participants’ subjective well-being and frequency of positive emotions over the past 7 days. Across PROMIS measures, a score of 50 represents the average adult population; scores ≥ 1 standard deviation worse than the general population (T-score ≥ 60 or ≤ 40) represent clinically elevated symptoms. The one-item Patient Global Impression of Change (PGIC), often used in chronic pain and pain rehabilitation settings [29,30], was administered post-series, asking participants to rate their overall change in well-being since the time they began the GVS, with responses from ‘very much worse’ (−3) to ‘very much improved’ (+3) [31].

Paper copies of a weekly progress sheet (see Supplementary Materials, S1) were administered at the beginning and end of each visit, inviting participants to rate their Overall Pain (1 = no pain; 10 = worst pain), Mental State (1 = poor; 10 = excellent), and Physical State (1 = poor; 10 = excellent), complete a pain heat-map, describe their symptoms, and provide written reflections on their experience during the session. Though this weekly progress sheet has not been formally validated, it has been utilized in previous program evaluations and for clinical care and documentation in chronic pain GMVs by several experienced clinicians [23].

All participants were offered the opportunity to participate in a 30 min post-GVS interview one month after its completion. A semi-structured interview guide was developed for this program, utilizing inclusive interview and purposive sampling techniques (see Supplementary Materials, S2) [32,33]. The lead qualitative investigator (TR) directed the interview guide’s development and training on interview protocol. Interviewers had additional training on in-depth semi-structured and unstructured interviewing. This training included synchronous mock interviews with feedback.

Two investigators (JK, JR) without prior contact with participants conducted audio-only interviews using Microsoft Teams (Version 1.8.00.21151, Microsoft, Redmond, WA, USA, accessed during fall 2025); audio was recorded through the desktop application Audacity (Version 3.7.5, Muse Group, Limassol, Cyprus, accessed during fall 2025). Any personally identifiable information shared during interviews was removed prior to being numerically coded and saved on a secure network drive. Recordings were transcribed with MAXQDA (Version 26.2, VERBI Software GmbH, Berlin, Germany, first accessed 5 May 2026) and reviewed to ensure no identifiable information remained before sharing with the independent qualitative investigators for formal analysis.

Participation in the program evaluation was voluntary. PROMIS-29, Global Impression of Change, and weekly progress sheets were integrated into the program without any incentives for completion. Completion of the additional PROMIS-Positive Affect survey was incentivized with a $50 gift card, and interview participants were given an additional $50 gift card. The TLC-HoW program evaluation initiative was reviewed by Hennepin Healthcare’s Institutional Review Board (IRB) (approval code: IRB-FY2024-870, 23 July 2024) and determined that it did not constitute human subjects research and, therefore, was not subject to IRB oversight.

### 2.5. Analysis

Based on attendance, the percentage of visits with ≥6 participants was calculated. Weekly progress sheet completion was calculated as a percentage of completed sheets divided by total attendance.

The analysis of preliminary program impact on participants utilized descriptive statistics (Mean (M); Standard Deviation (SD)) to assess participants’ reported changes on outcome measures over time. PROMIS-29 Physical and Mental Health Summary scores were calculated from raw domain scores for mean difference analysis at visits 4 and 8. PROMIS-Positive Affect mean difference analysis was conducted at visit 8. All participants who attended ≥3 visits and completed baseline and ≥1 follow-up PROMIS-29 survey were included in the mean difference analysis. Minimal Clinical Important Difference (MCID), with a threshold improvement of ≥3 units on the T-score scale, was employed to identify clinically meaningful improvements at the individual participant level [34,35,36] for all PROMIS scores except pain intensity. The average Patient Global Impression of Change (PGIC) was calculated across participants. All analyses were performed using Microsoft Excel.

Weekly progress sheets consisted of a journal-style prompt inviting participants to write about their present physical, mental, and overall state and their experience of the GVS. Raw data (responses to prompts) was transcribed using manual entry in Microsoft Excel (TR). If participant writing was illegible, an attempt was made to record and mark it with an “(il)”. Using a direct content analysis [37], inductive coding was employed. Codes were thematically grouped and defined. Then, a final review with general labels of frequency (high, medium, and low) was added.

Post-GVS interviews were examined through rapid qualitative analysis using a five-step model: (1) plan and prepare; (2) engage with data individually; (3) engage with data as a group; (4) collate learnings; and (5) summarize findings [38]. Two independent, experienced qualitative investigators (TR, KL) moved through each of the five steps (spring 2026). The lead qualitative investigator (TR) led the collaborative rapid analysis. Both investigators were trained in Qualitative Methodology, including historical framing, depth/breadth, reflexivity, and trustworthiness.

For step 1, investigators agreed on (line-by-line) segmentation and initial generation of each transcript. Initial segmenting was decided on in connection to previous work [39]: Skills Learned, Material Presented, Experience in Group, and Suggestions for Improvement. An Excel document was prepared with a tab for each segment, including “Insight/Note” and quotes. For step 2, investigators used personal techniques for the initial review of all transcripts. Patterns were identified as “Insight/Note”. For step 3, investigators met to discuss initial insights in segments. Saturation was not possible, as sample size and richness (i.e., conceptual depth) [40] did not provide in-depth phenomenological data. However, the qualitative investigators found that concepts did overlap in meaningful ways. To set a threshold of concepts to report, investigators found that most concept overlap happened in 3/6 interviews and minimal overlap occurred in 2/6. Therefore, investigators decided to keep concepts that emerged in half of the interviews. The decision to align overall themes by presence and notable remarks was made. For step 4, alignment of concept presence and notable statements was collected. For step 5, the team split final segments into five categories and one sub-category with examples. Investigators met for 2 h to confer on examples to use. Rapid thematic analysis was carried out in a team-based meeting structure where discussions on themes evolved in collaboration. There were no disagreements on themes; however, there were also no individually identified themes or codes to be tested in this fashion. This was not a coded or positivist method, but rather an exploratory review.

## 3. Results

**Figure 1:**
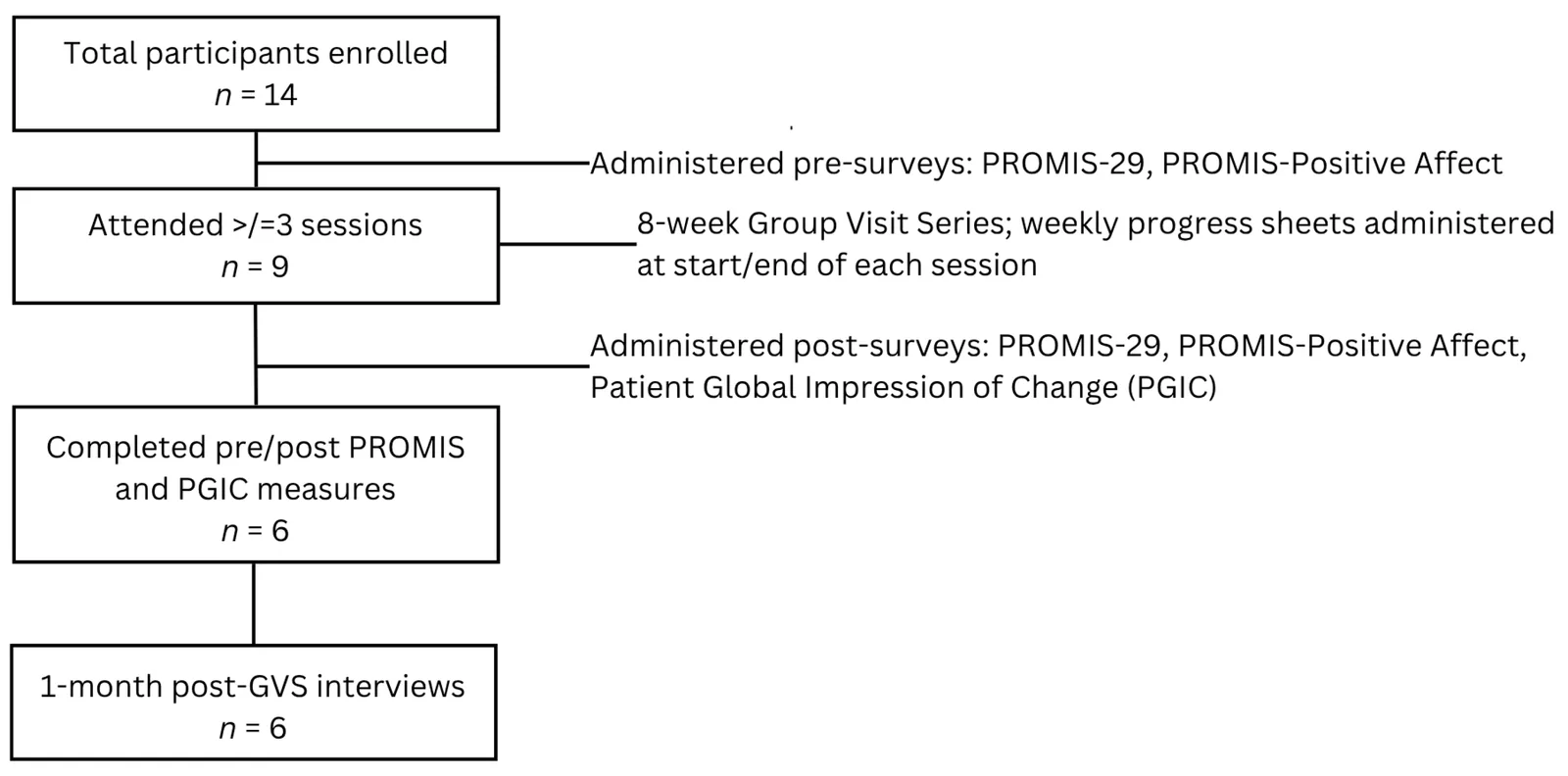
TLC-HoW opportunity youth flowchart.

Fourteen participants enrolled in the pilot program; nine attended ≥3 visits (indicating minimum participation). Of the 12 participants who enrolled prior to program interruption, 8 returned afterward, and 4 did not return to the host site after the interruption. Enrolled participants identified as Black (*n* = 10; 71.4%), White (*n* = 1; 7.1%), Hispanic (*n* = 1; 7.1%), Unknown/Declined to Answer (*n* = 2; 16.7%), Female (*n* = 8; 57.1%), and Male (*n* = 6; 42.9%). Detailed attendance reporting for the program is described elsewhere [26]. Figure 1 shows the participant flow throughout the program.

Feasibility benchmarks were met, with ≥6 participants being present at 87.5% of visits (see Supplementary Materials, Table S1) and a 96.4% weekly progress sheet completion rate from a total of 53 completed weekly progress sheets.

Regarding program sustainability, after completing the pilot GVS as part of the TLC-HoW initiative, the host site continued to offer the GVS due to positive participant feedback and requests for an ongoing series. In the 12 months following the pilot GVS, the host site has offered two additional GVS for which attendance was tracked. Organization A secured additional funding to continue the GVS at the host site and provided the staffing and resources to continue the program. Prior GVS participants volunteered to staff a table at recruitment events, informing other students about the program and encouraging them to engage. This proved to be an effective means of recruitment, as both subsequent GVS were filled to their site capacity of 12 participants with an average of 8 students per visit (range 4–10). The curriculum has also undergone modifications based on participant feedback, including the addition of a nutrition educator at each visit due to requests from students for more nutrition and culinary skills education. The GVS has been included in the students’ daily schedule, signaling to both students and trade instructors the priority the organization assigned to the program and removing a previous barrier to participation.

To explore program acceptability, a qualitative analysis examined two weekly progress sheet questions soliciting participant feedback about the program. In response to “What portion(s) of the group visit did you like and/or plan to try?”, the most frequent responses were: yoga (including specific postures, breathing, and meditation practices), talking/sharing/discussion, and drawing (final visit only). There were only 10 responses (out of 53 weekly progress sheets completed) to the question “Any suggestions for improvement or other topics?”, including calming music as the most frequent response, more art activities, different types of food, stretches, improvement of mental health and mental pain, and replacing AI with clipart.

Exploratory participant-reported outcomes from PROMIS-29, PROMIS Positive Affect and PGIC showed trends in an overall positive direction. Average visit 1 PROMIS-29 Physical and Mental Health Summary Scores (*n* = 6) were 45.7 and 43.9; visit 4 scores (*n* = 5) were 50.9 and 46.1, which improved to 49.2 (+3.5) and 47.6 (+3.7) at visit 8 (*n* = 6), respectively. Table 2 reports Visit 1, 4, and 8 PROMIS-29 mean domain scores. Average baseline scores for Anxiety and Depression reflected clinically elevated symptoms (≥1 standard deviation worse score than the general population (T-score ≥ 60 or ≤40)) [35]. All domains (Physical Function, Anxiety, Depression, Fatigue, Social Roles, Pain Interference, and Pain Intensity) except Sleep Disturbance showed numeric improvement by visit 8. Clinically Meaningful Improvement was most notable in Depression (6/6 respondents, 100%), Anxiety (5/6 respondents, 83.3%), and Social Roles (4/6 respondents, 66.7%).

The average PROMIS-Positive Affect score (*n* = 5) at baseline was 42.8, which improved to 46.1 (+3.3) at visit 8. Participants’ Global Impression of Change was +1.8 (*n* = 6).

On the weekly progress sheets, the average pre-visit Overall Pain score across the series was 3.0. Average scores for Mental and Physical State were 6.0 and 5.9, respectively. The average intra-visit change (pre-/post each visit) for Overall Pain increased by 0.7, and Mental and Physical State both improved by 1.1 and 0.7, respectively. Table 3 displays the average scores by visit (1–8) for Overall Pain and Mental and Physical State, followed by the average intra-visit change. Before and after visit themes, in response to the prompts “Describe how you are feeling before group today” and “Describe how you are feeling after group today,” are reported in Table 4.

In the 1-month post-GVS semi-structured interviews (*n* = 6), participants shared skills that they learned or remembered as well as reflections on the impact of the GVS experience on their day-to-day lives. The independent qualitative investigators identified four concepts that stood out through the interviews: Skills, Group Experience, Experience, and Improvement.

**Skills:** While there was a distinction between statements of skill acquisition (i.e., something they learned) and material (i.e., something they remembered doing), there was overlap between statements, so the concepts were clumped together. The concepts observed were: yoga, meditation, mental/emotional health, nutrition, sleep, and takeaway materials. Every participant reported some kind of ongoing benefit from yoga, meditation, mental/emotional health, or habits. Statements suggested improvements in well-being such as a calmer mind, reduced stress, and a sense of more space, either emotionally or in their schedule. Table 5 details the skill concepts by presence and includes a representative example.

**Group Experience:** In addition to the skills developed, four out of the six interviews commented on the social experience. Participants also talked about meeting people who understood their experience.

**Experience:** Participants described how the experiences within the GVS shifted their lifestyle, with 4/6 participants mentioning the benefit of building healthy habits and new routines.

**Improvement:** Participants were asked about what did not work for them in the GVS. The responses fell into three areas: (1) physical location, (2) personal alignment, and (3) additional recommendations. Physical location seemed to be important to three participants. Specifically, two participants recommended going outside for more fresh air and relaxation. Participants noted different skills or activities that did not align with their own individual experience. Additionally, some participants expressed a desire for more discussion time, one-on-one visits, and/or a deeper exploration of food’s relationship to pain. These improvement suggestions are noted as an element of the program’s acceptability and were utilized for ongoing program refinement.

## 4. Discussion

Organization A successfully adapted and implemented the 8-week pilot GVS, meeting or exceeding feasibility indicators observed through attendance and weekly progress sheet completion rates. Sustainability was seen by the continuation of the GVS beyond the pilot and growing interest among students and the host site. Acceptability was reflected in attendance, minimal suggested improvements, and interest in program continuation beyond the pilot. Participant-reported outcome measures demonstrated associations with generally positive changes across physical and psychosocial domains.

The curriculum adaptations from this pilot contrast with those of the other two organizations in the TLC-HoW program. Organizations B and C served rural adult populations, with curricula focused on chronic pain care [26]. Organization A’s focus on an urban opportunity youth population necessitated unique curricular adaptations. The decision to frame the GVS around stress and anxiety alongside physical pain was informed by the pediatricians’ clinical expertise and research that highlights the interconnected nature of mental/emotional health with physical symptoms and the multidimensional chronic pain experience [18,41,42,43]. This reframing of the GVS to include mental and emotional health alongside physical pain may have been an additional factor in its popularity and ongoing engagement.

Attendance and qualitative outcomes demonstrate the feasibility and acceptability of the program. The incorporation of sessions into the school day and their physical location at the residential employment-readiness center likely contributed to consistent attendance. The provision of dedicated time to complete the weekly progress sheets may have contributed to their high completion rates. Although the 7-week interruption was unplanned, the fact that all participants who returned to the center chose to continue the group points to the GVS’s feasibility and acceptability. Acceptability was also supported by the participants’ voluntary involvement in recruitment efforts for future GVS. Although prior participants requested to attend the GVS a second time, they could not be accommodated because the GVS were fully enrolled. This ongoing engagement points towards appreciation and acceptance of the GVS among this opportunity youth population at the residential employment-readiness center. While suggestions for improvement were minimal, participant interviews revealed that the inclusion of music, an outdoor experience, and more time for discussion may be worth considering for future programs.

Although exploratory, the qualitative and quantitative participant-reported outcomes from the pilot GVS align with adult chronic pain GMV and adolescent chronic pain studies. Though based on a small *n* value, the PROMIS-29 findings point to potential associations with clinically meaningful changes in aspects of chronic pain and improved mental health and social functioning, which are established resilience factors [41,44]. Although not matched exactly, the multidimensional biopsychosocial domains of PROMIS-29 are all represented in the lifestyle-related topics covered throughout the GVS (and in the original TLC-HoW chronic pain GMV curriculum), which may be a potential contributor to the improved scores seen between visit 1 and visit 8. Interestingly, Sleep Disturbance showed increasing (worsening) numeric scores from visit 1 to visit 4 and visit 8; however, 33% of respondents met the MCID threshold. This dichotomy represents a wide spread of scores within the small *n* value. Further potential explanations for the worsening of some scores are various external factors that the participants experienced during that particular span of weeks (returning to the residential center after the 7-week interruption, family/social stressors, etc.) and/or the modification of the dedicated session on sleep to include mood and pain. The unplanned 7-week interruption between visits 3 and 4 may have potentially worsened scores due to the stress and uncertainty during that time.

As reported previously, the inclusion of a PROMIS-29 assessment midway through an 8-week GMV may provide insight into the trajectory of participants’ experience [23]. In the case of this program, the PROMIS-29 administered at visit 4 occurred after the 7-week interruption, yet still demonstrated improvements that reached MCID for a portion of the participants. Due to numerous variables, including the small number of participants and the lack of a control group, these improvements cannot be attributed to the program intervention but are still notable, with scores in some domains trending similarly at visit 8. Future studies could consider comparing variable intervention lengths to determine an effective “dose” of GVS participation.

The weekly progress sheet’s quantitative, intra-visit ratings describe improvements in participants’ weekly self-reported Physical and Mental States, while Overall Pain scores worsened slightly. These intra-visit data contrast with PROMIS-29 results showing clinically meaningful improvements in Pain Interference, as well as modest improvements in Physical and Mental Health Summary Scores. The contrast of slightly worsening pain ratings next to slightly improving subjective Physical and Mental state ratings was found in another study of chronic pain in youth [18]. Potential explanations for the differing pain ratings may be associated with the differences in the timing of the assessments: weekly progress sheets queried pain levels before and after each session, while the PROMIS-29 queried pain intensity and interference over the prior 7 days, measured at three different time points. Intra-visit pain scores could reflect physical discomfort from the session itself (chairs, duration of sitting, etc.), specific activities occurring prior to or during GVS sessions, and/or increased awareness of bodily sensations fostered through the practices within the GVS sessions. This dichotomy also raises the question of how Overall Pain, Physical and Mental states may or may not be interrelated.

In particular, the qualitative interviews highlight the value of the group environment, peer connection, the importance of shared experiences, mental health support, and appreciation for experiential skill-building in yoga, meditation, and nutrition. Other chronic pain GMV studies in adult populations support the importance of group processing, social connection, and experiential activities as participants’ favorite aspects of the group experience [23,45,46]. These qualitative findings alongside the changes in PROMIS-29 domains further support the interconnected nature of mental health, chronic pain, lifestyle factors and social connection.

Self-regulation abilities have been suggested as a potential mechanism for improved chronic illness management in adolescent populations [47]. Qualitative analyses suggest that offering nervous system regulation education/training plus mindfulness-based interventions such as meditation and yoga in this curriculum may have fostered skills or practices which influenced the improved mental health symptoms suggested by the PROMIS-29 scores. However, this interpretation should be considered hypothetical given the small *n* value and lack of control conditions.

To summarize, this program evaluation’s findings could be considered within three levels of inference: observed findings, interpretation, and hypotheses. Observed findings from this pilot program evaluation included attendance, participant feedback, weekly progress sheet responses, PROMIS and PGIC results, and continuation of the GVS beyond the pilot. Interpretation included feasibility, acceptability, and preliminary associations indicating potential program benefit. Hypotheses include the possible associations between the GVS intervention and positive trends in participant-reported mental health and pain experiences.

### 4.1. Lessons Learned

The successful piloting of this program yielded important lessons regarding practical implementation. In addition to adapting the GVS content for this unique population of adolescents, the pediatricians made iterative delivery changes, including greater emphasis on experiential and discussion opportunities and limiting didactic teaching to <10 min segments. Participants preferred asking questions about the topics and referencing their own or their peers’ experiences over a more traditional classroom-type approach. These observations follow the adult learning theory principles of new material being relevant to existing knowledge or experience, practical, and goal-oriented [48]. The utility of experiential learning for both adolescents and adults is well established, with a recent meta-analysis indicating additional improvements in pro-social behaviors, empathy, and wellbeing through such interventions [49].

Including the host site’s pediatrician in the initial TLC-HoW training program allowed for the GVS to continue beyond the TLC-HoW grant period. Host-site support was critical to the program’s success and sustainability of the program. This support increased as the students spoke highly of their experience, facilitating continuation of the GVS beyond the initial pilot funding. Organization A fully resourced the GVS program; host-site support included classroom use for the GVS, communication with participants outside of the GVS, and course schedule management to allow for GVS attendance. The alignment of the GVS program with the employment-readiness center’s goals to improve the health of students in support of their long-term employability and overall success was another factor likely driving sustainability. Participant suggestions for improvement (outdoor activities, more time for sharing, inclusion of different topics, etc.) continue to be considered.

### 4.2. Limitations and Future Directions

This program evaluation has important limitations. As a single-site, uncontrolled pilot program with a small number of participants, these results should be interpreted with caution, and no causal claims can be inferred. It is unclear to what extent the 7-week interruption in the GVS between visits 3 and 4 may have influenced participant-reported outcomes. The two pediatricians leading the GVS had no contact with participants during the 7 weeks they were away from the center. On the one hand, the disruption may have interrupted the progressive skill development trajectory or group cohesion, potentially reducing program impact. Conversely, the shared external threat may have fostered group cohesion and underscored the relevance of the program’s approach and tools. The fact that all participants who returned to the center also resumed the GVS following the interruption is mirrored by ongoing interest among students in subsequent GVS beyond the pilot, suggesting this GVS program is meeting a need for students.

Participants’ baseline symptoms and clinical conditions were not collected beyond their initial self-report on weekly progress sheets and other self-reported outcome measures, limiting interpretation of the program’s impact on individual participants. Additional limitations or confounding factors include potential selection bias, social desirability biases in various aspects (attendance, weekly progress sheet completion, and interviews), the influence of incentives on participant-reported outcomes, and the lack of a control group. The small sample size for qualitative outcomes meant that saturation was not achieved, further limiting the generalizability of qualitative themes. Due to the program evaluation design, program timeline, and staffing capacity, participants did not have the opportunity to verify interpretations of their interview data or feedback.

Logistical issues influenced the PROMIS-29 response rate; the use of paper surveys only allowed people who physically attended visits 1, 4, and 8 to complete measures, narrowing the comparison pool. Some participants did not return after the interruption and/or were lost to follow-up. Physical copies of survey measures were manually entered into REDCap for analysis; electronic survey capture could have improved receipt of outcomes from more participants, as well as the benefits of time, accuracy, and full anonymity. While the weekly progress sheet has been used in other studies [23,24,25,39], it has not been formally validated. No long-term follow-up data has been collected, thus limiting assessment of program impact or causal relationships. Future research should investigate multiple sites, larger subject pools, comparison groups, and follow-up data collection.

## 5. Conclusions

An eight-visit GVS to support chronic pain and mental health was piloted with opportunity youth at an urban, residential employment-readiness center. Within the limitations of this small, uncontrolled program evaluation, implementation of the pilot GVS, adapted from an adult whole-person chronic pain GMV curriculum, was feasible and has led to sustainable continuation beyond the pilot. Acceptability of the GVS was demonstrated by high engagement despite an unplanned program interruption, positive participant feedback, minimal suggestions for improvement, and continued interest in program participation. The participant-reported outcomes offer preliminary signals that the GVS curriculum could be associated with mind–body skill development, positive peer connections, improved mental health, and decreased pain interference, with some outcomes reaching clinically meaningful thresholds. Further study, with a larger subject pool, control groups, and more standardized outcome collections, is needed to determine causal associations. This GVS could serve as a model for a wider opportunity youth population alongside other skill-building programs in this unique population of young adults.

## Figures and Tables

**Table 1:** Comparison of curriculum topics from the original Center for Teaching, Learning, and Coordination—Heals on Wheels (TLC-HoW) program with the Group Visit Series (GVS) for opportunity youth.

Week/Visit	TLC-HoW GMV Curriculum Topics	Organization A GVS Topics
1	Science of chronic pain	Stress, pain and anxiety
2	Stress response and pain	Thoughts and emotions
3	Thoughts and emotions	Food and inflammation
4	Food and inflammation	Movement and the outdoors
5	Integrative modalities (yoga, acupuncture, chiropractic, massage)	Yoga
6	Movement and pacing	Sleep, relaxation, mood and pain
7	Sleep, rest, and relaxation	Creativity and community
8	Spirituality and community	Wrap-up and next steps

Note: GMV = Group Medical Visit; GVS = Group Visit Series.

**Table 2:** Participant-reported outcomes PROMIS-29 average domain score and percentage of participants demonstrating clinically meaningful improvements (*n* = 6).

Domain	Visit 1 (*n* = 6) Mean (SD)	Visit 4 (*n* = 5) Mean (SD) +/− Mean Difference	Visit 8 (*n* = 6) Mean (SD) +/− Mean Difference	CMI ** (%)
Physical Function *	45.8 (6.5)	51.5 (7.4)	48.8 (7.1)	50.0
		+5.7	+3.0	
Anxiety	62.7 (6.5)	57.9 (10.1)	54.7 (9.3)	83.3
		−4.8	−8.0	
Depression	61.2 (6.6)	53.4 (12.3)	48.9 (9.5)	100.0
		−7.8	−12.3	
Fatigue	54.6 (9.3)	52.4 (8.5)	54.4 (8.3)	50.0
		−2.2	−0.2	
Sleep Disturbance	54.6 (11.9)	55.9 (8.7)	58.1 (8.9)	33.3
		+1.3	+3.5	
Social Roles *	51.8 (10.3)	48.7 (6.8)	54.0 (6.1)	66.7
		−3.1	+2.2	
Pain Interference	53.8 (9.5)	52.9 (7.9)	50.8 (7.9)	50.0
		−0.9	−3.0	
Pain Intensity	4.2 (2.5)	3.0 (1.9)	2.8 (1.3)	n/a
		−1.2	−1.4	

Note: PROMIS-29 average domain scores at visit 1, 4, and 8, and the percentage of participants reporting Clinically Meaningful Improvement (CMI) for each domain; n/a = not applicable to the single-item pain intensity rating. * Increased scores for these domains indicate an improvement in symptoms. ** Percentage of participants demonstrating Clinically Meaningful Improvement (CMI) of ≥3 points at visit 8 for each domain.

**Table 3:** Weekly progress sheet scores for Overall Pain, Physical State and Mental State.

**Average Scores from Weekly Progress Sheets**				
**Week**	**Attendance**	**Overall Pain**	**Mental**	**Physical**
1	12	3.375	4	4
2	9	3.86	5.43	5.571
3	4	4.33	7	7.33
4	6	2.33	5.17	5.33
5	7	2.33	4.5	4.5
6	6	2.6	7.4	6
7	8	2.63	7.125	7.625
8	8	2.12	7.25	7.13
**Average**	**7.5**	**2.95**	**5.98**	**5.94**
**Weekly progress sheet change in State scores pre-/post- each GVS session**				
**Week**	**Attendance**	**Overall Pain**	**Mental**	**Physical**
1	12	0.625	0.83	0.33
2	9	0.423	0.86	−0.28
3	4	−1	1.33	0.33
4	6	1	1.5	2.17
5	7	−0.33	1.25	1.25
6	6	0.4	0.77	0.5
7	8	0.25	1	0.5
8	8	0	1.125	0.5
**Average**	**7.5**	**0.71**	**1.08**	**0.66**

Note. Week 3 had low participation; some post scores missing from weeks 1 and 5. Overall Pain scores rated 1–10 (10 being worst pain); Mental and Physical State rated 1–10 (10 being best); improvement in Overall Pain is negative whereas improvement in Mental and Physical State is positive.

**Table 4:** Weekly progress sheet qualitative data.

Prompt	Theme	Persistence	Definition	Example
Describe how you are feeling before group.	Physical sensation	High	Description of sensation or pain.	“lot of pain through my body”; “stiff”; “tense”; “stomach”
	Fatigue/calm	High	Expression or description of low energy.	“Exhausted with outside work”; “cloudy”; “calm”; “chill, tired”
	Concern	High	Expression of stress, overwhelm, or worry.	“anxious”; “a little irritable, a little stressed”; “overthinking”; “nervous”
	Brief and neutral	Mid	Short single-word phrases.	“ok”; “fine”; “good”
	Hopeful	Low	Expression of progress or desire to learn.	“good, proud for meeting goals”; “tired, determined, focus”
Describe how you are feeling after group.	Calm	High	Descriptions of reduced arousal.	“calm, relaxed, happy”; “calmer”; “tired but in a good way”
	Mixed impact	Mid	When describing symptom change, it was mixed.	“physically the same”; “still tired and cold”; “more tired, feeling sleep”; “a tiny bit better”;
	Motivated	Mid	Expressions of inspiration and wanting to use skills.	“motivated”; “happy”; “more mindful about my intake”; “happier”
	Connected	Low	Expressions of social connection.	“happy to be with everyone”; “collective”

**Table 5:** Skill concepts by presence in semi-structured qualitative interviews with participants in the group visit series (*n* = 6).

Concept	Presence	Example
Yoga	5/6	“the yoga mat being like having one of those kind of just gives me another reason to like, I wasn’t stretching, but like make sure I do push ups either in the morning when I wake up or at night before I go to bed. It’s just like there for a reason. I might as well use it. So yeah, make sure I do some push ups before work when I wake up.”
Meditation	5/6	“meditating. I do that every once in a while because like being in the school that I am is just like, my brain is constantly processed, like 1,000,000,002 things a day being able to like sit down and quiet and just let thoughts pass became very important. It’s like a clearer mind, really. It’s just I’m not as overwhelmed or overstimulated anymore. I have something to fall back, I can just.”
Mental/Emotional Health	4/6	“I liked how they gave me a couple, like, tips on, like, for mental health experience, like ways for me to help, like activities and more like, um, and more like, um, non stress activities because I know sometimes I get a lot of crankiness, but during that um, program really helped me like loosen stress and finding other ways to like let loose or like, not go too mad.”
Nutrition	4/6	“I’ve been eating a few of the snacks that they’ve are recommended for inflammations. But I never knew that was like the perks of eating those things. So, um, that helped. And I also learned that there was a few more snacks that I could be eating to deal with those issues.”
Sleep	3/6	It was the sleep mask that we had got from it. I found out that it was actually kind of help with sleep.
Takeaway Materials	3/6	“sleep mask”; “aromatherapy”; “handouts”

Note. Presence was mutually decided per interview with no discrepancy. Quotes were decided on to serve as exemplars, but statements in interviews varied in length.

## Data Availability

The dataset for this study includes sensitive and confidential information, such as patient data; therefore, the dataset cannot be shared.

## Supplementary Materials

The following supporting information can be downloaded at: https://www.mdpi.com/article/10.3390/healthcare14213567/s1. S1: Weekly Progress Sheet; S2: Semi-Structured Interview Guide for Qualitative Post-Group Visit Series (GVS) Interviews; Table S1: Attendance reporting for GVS.
