# Supplementary material for: A Pilot Group Visit Series to Empower Opportunity Youth: Building Skills for Managing Pain, Anxiety, and Distress
Source: Healthcare (Basel). 2026 Oct 22;14(21):3567. doi: 10.3390/healthcare14213567 (PMC13762784; doi:10.3390/healthcare14213567)
Supplement: Supplementary file 1 [file healthcare-14-03567-s001.zip › Semi-Structured Interview Guide for Qualitative Post-Group Visit Series (GVS) Interviews (S2).pdf]

	**Center for Teaching, Learning, and Coordination - Heals on Wheels (TLC-HOW)**<br>**INTERVIEW** ID: _____________________ Date: ____
**Introduction to the Interview**	Hi, my name is _____, and this is _____. We are part of an integrative health team at Hennepin Healthcare. Thank you again for agreeing to do this interview with us today. It should take about 30 minutes.<br><br>We are conducting these interviews to improve group health visits like the one you were a part of. It's important that you know that I am not a medical doctor, and this is not a clinical assessment. In fact, you don't have to tell us about your symptoms at all – instead, we really want to know about your experience. There are no "right or wrong" answers and again, you don't have to tell us about your symptoms. Participation is totally voluntary. If you want to take a break or even stop, just let me know.<br><br>We would like to record this interview and will be taking notes. We will not use your name or any identifiable information in our transcriptions or notes. Information from program evaluations like this is always private and confidential.<br><br>If it is ok, I will start the tape recorder.<br><br>[START TAPE RECORDER, VERBALLY RECORD: DATE & TIME OF INTERVIEW – ID]<br>Starts: __________ (H) __________ (M) __________ (AM/PM<br>*Do you have any questions for me before we begin?*
**Experience in Group Visit**	Tell me about your experience at the group visit<br>*What do you remember the most?*<br>*What continues to come up for you since participating?*<br><br>Tell me about any skills or habits that you learned during the group visit that you still do.<br>*What skills or habits do you use?*<br>*What skills or habits have you tried?*<br>*What skills or habits do you not use and want to?*<br>*What didn't work?*<br><br>Tell me your overall thoughts/opinions of the entire experience<br>*What could we do to improve the experience?*<br>*Issues relevant to the content of group sessions?*
**Closing**	Is there anything else that you'd like to share?<br><br>Ends: __________ (H) __________ (M) __________ (AM/PM)
