# Supplementary material for: A Pilot Group Visit Series to Empower Opportunity Youth: Building Skills for Managing Pain, Anxiety, and Distress
Source: Healthcare (Basel). 2026 Oct 22;14(21):3567. doi: 10.3390/healthcare14213567 (PMC13762784; doi:10.3390/healthcare14213567)
Supplement: Supplementary file 1 [file healthcare-14-03567-s001.zip › Supplemental Materials Table S1_ Attendance reporting for GVS - english.pdf]

Supplemental Materials Table S1
Attendance reporting for GVS.

Group Visit Series (GVS) Session	Number of Participants
GVS1	12
GVS2	9
GVS3	4
GVS4	6
GVS5	7
GVS6	6
GVS7	8
GVS8	8
