# Supplementary material for: A Pilot Group Visit Series to Empower Opportunity Youth: Building Skills for Managing Pain, Anxiety, and Distress
Source: Healthcare (Basel). 2026 Oct 22;14(21):3567. doi: 10.3390/healthcare14213567 (PMC13762784; doi:10.3390/healthcare14213567)
Supplement: Supplementary file 1 [file healthcare-14-03567-s001.zip › Weekly Progress Sheet (S1).pdf]

Integrative Health
Heals on Wheels

HennepinHealthcare

ID

Date

Pre-Session Survey

Heartrate

Blood Pressure

Overall

In general, how is your pain today? *circle below*

no pain 1 2 3 4 5 6 7 8 9 10 worst pain

Mental state

How would you rate your overall mental state?

Poor 1 2 3 4 5 6 7 8 9 10 Excellent

Physical condition

How would you rate how you feel physically today?

Poor 1 2 3 4 5 6 7 8 9 10 Excellent

Describe how you are feeling before group today.

What is something you have done over the past week that eases your pain?

Tell us about your pain

Mark an X on the parts of the body in which you've experienced pain in the past week.

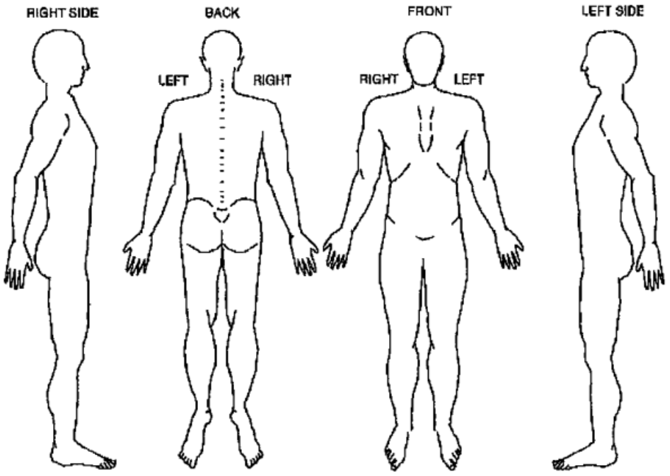

Tell us about your symptoms

Circle the descriptions below of your pain this week.

- Sharp
- Tension
- Tightness
- Aching
- Stabbing
- Shooting
- Burning
- Numbness
- Pressure
- Weakness
- Other

HennepinHealthcare

Integrative Health
Heals on Wheels

Post-Session Survey

Heartrate		Blood Pressure	

Overall

In general, how is your pain after this group? *circle below*

no pain 1 2 3 4 5 6 7 8 9 10 worst pain

Mental state

How would you rate your overall mental state?

Poor 1 2 3 4 5 6 7 8 9 10 Excellent

Physical condition

How would you rate how you feel physically today?

Poor 1 2 3 4 5 6 7 8 9 10 Excellent

Describe how you are feeling after group today?

What is your goal this week?

What portion(s) of the group visit did you like and/or plan to try?

Any suggestions for improvement or other topics?

If you have any specific needs for your doctor, please describe them here.
